# LAPTM4B is a novel diagnostic and prognostic marker for lung adenocarcinoma and associated with mutant EGFR

**DOI:** 10.1186/s12885-019-5506-7

**Published:** 2019-04-02

**Authors:** Lu Wang, Yue Meng, Qing-Yun Zhang

**Affiliations:** Department of Clinical Laboratory, Key Laboratory of Carcinogenesis and Translational Research (Ministry of Education), Peking University School of Oncology, Beijing Cancer Hospital and Institute, 52 Fucheng Road, Haidian District, Beijing, 100142 China

**Keywords:** LAPTM4B, Lung adenocarcinoma, Epidermal growth factor receptor, Prognosis, Biomarker

## Abstract

**Background:**

Lysosomal-associated protein transmembrane-4 beta (LAPTM4B), a novel oncogene, promotes tumorigenesis and may be a potential prognostic biomarker in several cancers. This study was to determine the clinical significance and biological roles of LAPTM4B in lung adenocarcinoma (LAC).

**Methods:**

LAPTM4B expression was analyzed by immunohistochemistry (IHC) of 63 LAC tumors. Serum levels of LAPTM4B were measured by enzyme-linked immuosorbent assays (ELISA). The study included untreated group (*n* = 216), chemotherapy group (*n* = 29), chemotherapy efficacy group (*n* = 179), EGFR-TKIs group (*n* = 57) and 68 healthy controls. Statistical analysis was performed to explore the correlation between LAPTM4B expression and clinicopathological parameters in LAC. Kaplan-Meier analysis was performed to assess the prognostic significance of LAPTM4B in LAC. In vitro assays were performed to assess the biological roles of LAPTM4B in LAC cells. Western blotting assays were examined to identify the underlying pathways involved in the tumor-promoting role of LAPTM4B.

**Results:**

We found LAPTM4B was upregulated in LAC tissues and high LAPTM4B expression was significantly correlated with poor prognosis. Serum LAPTM4B levels were significantly decreased after chemotherapy. Patients in invalid response group showed higher LAPTM4B levels than the valid response group. Overexpression of LAPTM4B promoted, while silencing of LAPTM4B inhibited proliferation, invasion and migration of LAC cells via PI3K/AKT and EMT signals. LAPTM4B expression level was associated with epidermal growth factor receptor (EGFR) gene mutations. In addition, LAPTM4B plays important roles in EGFR-promoted cell proliferation, migration and invasion and gefitinib-induced apoptosis.

**Conclusions:**

Collectively, our data propose that LAPTM4B may be a cancer biomarker for LAC and a potential therapeutic target which facilitates the development of a novel therapeutic strategy against LAC.

**Electronic supplementary material:**

The online version of this article (10.1186/s12885-019-5506-7) contains supplementary material, which is available to authorized users.

## Background

Lung cancer is the leading cause cancer-related death worldwide and there are approximately 85% of lung cancer being classified as non-small cell lung cancer (NSCLC) [1]. Lung adenocarcinoma (LAC) is currently the main histological subtype of NSCLC and the average 5-year survival rate of NSCLC patients remains lower than 15% [2]. Early detection and lobectomy could significantly improve survival for early stage of lung cancer [3], but for late stage patients, lack of sufficient therapeutic methods resulted in cancer exacerbation. Currently, the widely used diagnostic markers for lung cancer are pro-gastrin-releasing peptide (ProGRP), neuron-specific enolase (NSE), carcinoembryonic antigen (CEA), and cytokeratin 19 fragment (CYFRA21-1) [4]. However, these tumor markers display relatively low sensitivity and specificity, so their clinical applications are limited [5, 6]. Thus, it is crucial for identification of new targets for diagnosis and monitoring treatment.

Lysosome-associated protein transmembrane-4 beta (LAPTM4B), a novel oncogene, was first identified in human hepatocellular carcinoma (HCC). LAPTM4B is tetratransmembrane lysosomal protein that is overexpressed and associated with poor prognosis in various malignancies including breast cancer, gallbladder cancer, ovarian cancer, HCC, gastric cancer and cervical cancer [7–15]. In addition, LAPTM4B has been reported to promote proliferation and metastasis of tumor cells, resist apoptosis, initiate autophagy and assist drug resistance [16]. Recent studies showed that LAPTM4B was elevated in NSCLC and its overexpression was an independent factor in NSCLC prognosis [17, 18]. However, the clinical significance of LAPTM4B in LAC and the role of this oncogene in malignant phenotype and cell signaling remain unclear.

In this study, we first demonstrated that LAPTM4B level was significantly elevated in LAC tissues as wells as serum samples, and indicated poor survival. Further functional assays showed that LAPTM4B promoted the oncogenic phenotypes of LAC cells in vitro via PI3K/AKT and EMT signals. In addition, LAPTM4B expression level was found to be associated with EGFR gene mutations and could be influenced by mutant EGFR in LAC. Taken together, we suggest that LAPTM4B is related to LAC progression and might be a potential biomarker and theraputic target for LAC.

## Methods

### Cell lines and cell culture

Human bronchial epithelial BEAS-2B (CRL-9609) cells and lung adenocarcinoma cell lines A549 (CCL-185), H1975 (CRL-5908), HCC827 (CRL-2868) and H1299 (CRL-5803) were obtained from American Type Culture Collection (ATCC, Manassas, VA, USA). The PC9 cell line was preserved by our laboratory. These cell lines were authenticated by short tandem repeat analysis (STR) by Beijing Microread Gene Technology Co., Ltd. (Beijing, China) in August, 2017. All the cell lines were confirmed without mycoplasma contamination. All the cell lines were maintained in RPMI-1640 supplemented with 10% FBS (Gibco, US) and antibiotics (100 μg/mL streptomycin and 100 units/mL penicillin) and cultured at 37 °C in a humidified incubator with 5% CO_2_.

### Tissue specimens

The total 63 LAC tissue samples used in this study were histopathologically diagnosed and surgically treated in Beijing Cancer Hospital between 2013 and 2014. Survival information was available for all patients until March 4, 2018. This study was approved by the Ethics Committee of Beijing Cancer Hospital and written informed consent was obtained from all participants.

### Immunohistochemistry (IHC)

Paraffin-embedded samples were cut into 4 μm and stained with H&E for tumor confirmation. Selected sections were immersed in 0.01 mol/L citrate buffer (PH 6.0) and incubated with and anti-LAPTM4B polyclonal antibody (Bioss Inc., bs-6542R) at 4 °C overnight. Stained tissue sections were evaluated separately by two pathologists without any knowledge of the clinical parameters. Staining was scored according to the previous criteria [19].

### Serum samples

Between September 2017 and January 2018, 216 serum specimens with histopathologically confirmed lung adenocarcinoma were enrolled as the LAC group. None of the patients had received adjuvant chemotherapy, immunotherapy, or radiotherapy before. In addition, 68 healthy controls including 28 males and 40 females, with a mean age of 40 years (range from 23 to 61), were chosen at the Medical Examination Center of Beijing Cancer Hospital. In addition, 29 LAC patients having received chemotherapy were enrolled in the chemotherapy group and their blood samples were collected before and after treatment, respectively. 179 LAC patients who were undergoing their chemotherapy cycles in Beijing Cancer Hospital were selected. Moreover, 57 LAC patients harboring EGFR mutations and having received EGFR-TKI treatment were enrolled. A CT scan was performed to assess the tumor size prior to initiating treatment and was repeated every 2 months. The treatment effect was assessed based on response evaluation criteria in solid tumors (RECIST) guidelines [20].

### Enzyme linked immunosorbent assay (ELISA)

A total of 5 mL peripheral venous blood was obtained and then centrifuged to collect serum. Serum samples were stored at − 80 °C for further use. The serum LAPTM4B was detected using quantitative human LAPTM4B sandwich enzyme immunoassay kits (LifeSpan BioSciences, Inc) following manufacturer’s instructions. The ELISA readings were measured at 450 nm in a microplate reader.

### Western blot analysis

The western blot assay was performed according to the previous description [19]. All the antibodies used for western blot analysis are shown in Additional file 1: Table S1.

### siRNAs and plasmid transfection

Stable LAPTM4B overexpression (GV358, Ubi-MCS-3FLAG-SV40-EGFP-IRES- puromycin) and the corresponding negative control cells were created through lentivirus infection. They were constructed by GeneChem (Shanghai, China). The specific LAPTM4B and EGFR siRNAs were synthesized by RiboBio (Guangzhou, China) and the sequences are shown in Additional file 2: Table S2. Transfection was performed with Lipofectamine 3000 reagent (Invitrogen, CA, USA) according to the manufacturer’s instructions.

### Cell proliferation assay

The Cell Counting Kit-8 (CCK-8; Dojindo, Kumamoto, Japan) was used to access cell viability. Cells (1 × 10^3 /well) were seeded into 96-well plates and each condition was repeated in triplicate. All cells were incubate for 5 days and 10 μL CCK-8 solution was added to each well at each indicated time point. Then the results were measured at 450 nm using a microplate reader according to the manufacturer’s instructions.

### Colony formation assay

Cells were plated in 60-mm dishes (500 cells/well). After incubation for 14 days, the colonies were fixed in 4% formaldehyde and stained with 1% crystal violet. Finally, positive colony formation (> 50 cells/colony) was counted under a microscope. All experiments were repeated in triplicate wells.

### In vitro cell migration and invasion assay

In vitro cell migration and invasion assay were performed following the previous study [19]. For the invasion assay, the cells were incubated for 24 h in the upper chamber coated with a mixture of serum-free medium and Matrigel, but for the migration assay, the cells were incubated for 12 h at 37 °C.

### Annexin V apoptosis assay

The HCC827 cells were incubated with 0.01 μM Gefitinib or DMSO and collected after 48-h incubation. Apoptosis was analyzed using an PE-Annexin V Apoptosis Detection Kit (BD Biosciences) with FACSCalibur flow cytometer (BD Biosciences).

### Statistical analysis

The statistical analyses were evaluated using the Statistical Software Package for the Social Sciences (SPSS software version 19.0, SPSS) and *P* < 0.05 was considered statistically significant. Measurement data was expressed as median (interquartile range, IQR) when the data did not meet the normal distribution. Associations between LAPTM4B expression and clinicopathological characteristics of LAC tissues were analyzed using the chi-squared test. Survival curves were plotted using the Kaplan-Meier method and compared using the log-rank test. Mann-Whitney *U* test was used for comparisons of two independent groups. Analysis of variance (ANOVA) was used to compare the differences among three groups.

## Results

### The expression of LAPTM4B in LAC tissues and correlation with prognosis

**Fig. 1:**
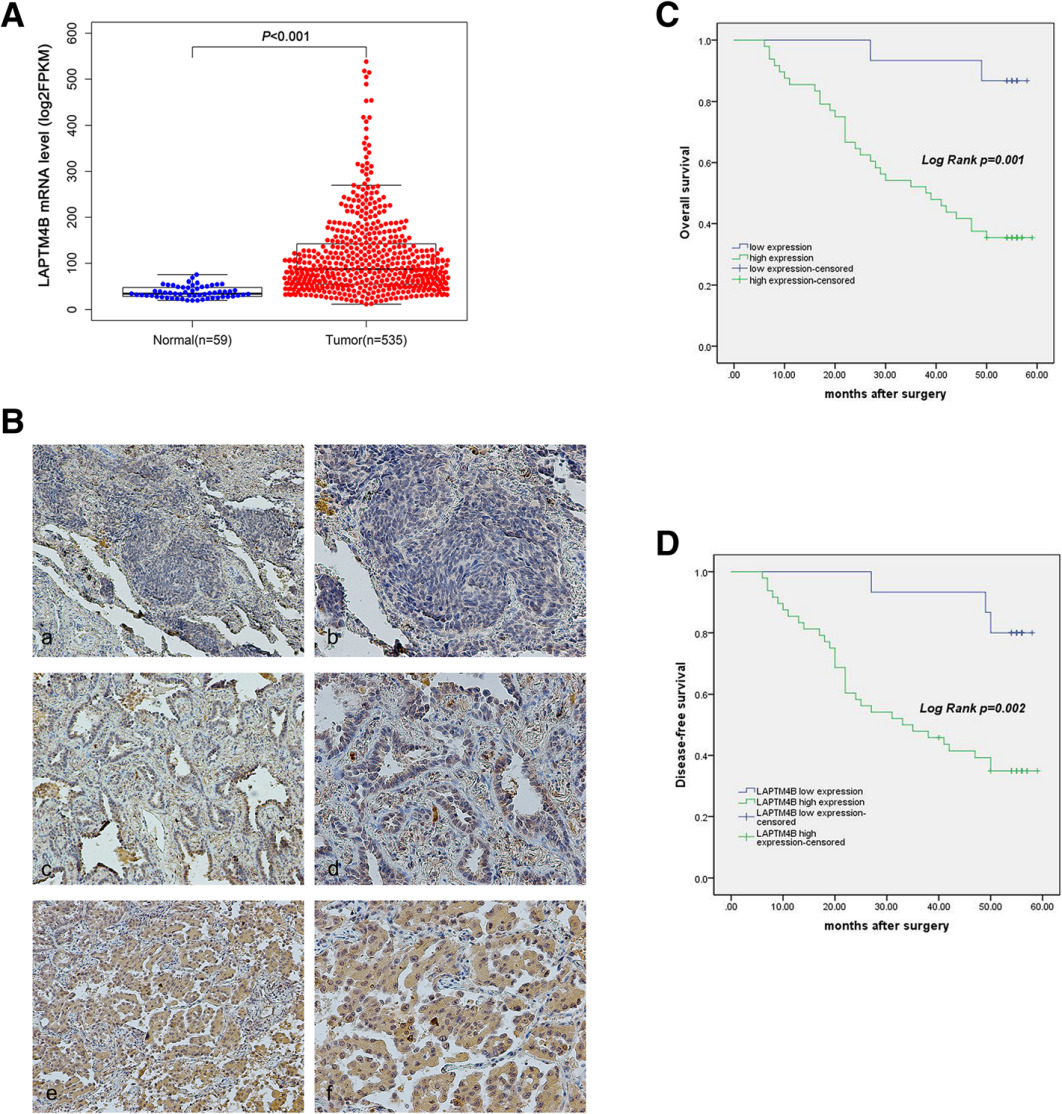
High expression of LAPTM4B in LAC tissues and correlates with poor patients survival. **a** The average expression level of LAPTM4B in patients with LAC with gains (amplification) was higher than those without gains in The Cancer Genome Atlas (TCGA) database. Each bar represents the median values±quartile values. **b** Immunohistochemical analysis of LAPTM4B expression in LAC patients. **a** and **b** Negative expression of LAPTM4B. **c** and **d** Low expression of LAPTM4B. **e** and **f** High expression of LAPTM4B. **a**, **c**, **e**. Original magnification × 100; **b**, **d**, **f**. Original magnification × 200. C and D Kaplan-Meier overall survival and disease-free survival curves for patients with LAC stratified by high and low expression of LAPTM4B

We first analyzed LAPTM4B mRNA expression levels in LAC tissue from TCGA (The Cancer Genome Atlas) database and revealed that LAPTM4B was upregulated in LAC tissues compared with normal tissue samples (Fig. 1a) .

In addition, we sought to characterize LAPTM4B expression in 63 LAC specimens in the context of various clinicopathological variables including patients outcome (Fig. 1b). The IHC assay showed that high expression of LAPTM4B was observed in 48/63 (76.2%) LAC tissue samples. In addition, the expression levels of LAPTM4B were positively correlated with advanced clinical stages, lymph node metastasis and EGFR mutations. However, no statistically significant correlations were identified between the LAPTM4B levels and other clinicopathological characteristics including gender, age, smoking, hypertension depth of infiltration, tumor size and K-ras mutations (Table 1). Kaplan-Meier survival analysis revealed that patients with high LAPTM4B expression exhibited shorter overall survival and disease-free survival compared to those with LAPTM4B low expression (Fig. 1c, d).

**Table 1:** Associations between the expression levels of LAPTM4B and clinicopathological characteristics in 63 LAC patients

Characteristics	Number	Expression of LAPTM4B		*P* value
		Low(*n* = 15)	High(*n* = 48)	
Gender				
Male	21(33.33%)	5	16	1.000
Female	42(66.67%)	10	32	
Age				
≤ 60	20(31.75%)	3	17	0.349
> 60	43(68.25%)	12	31	
Smoking				
No	48(76.2%)	12	36	1.000
Yes	15(23.8%)	3	12	
Hypertension				
No	41(65.1%)	10	31	0.883
Yes	22(34.9%)	5	17	
Depth of infiltration				
T1 + T2	59(93.65%)	15	44	0.564
T3 + T4	4(6.35%)	0	4	
Clinical stages				
I + II	50(79.37%)	15	35	0.027
III + IV	13(20.63%)	0	13	
Lymph nodes status				
No	42(66.67%)	14	28	0.012
Yes	21(33.33%)	1	20	
Tumor size				
< 3 cm	43(68.25%)	13	30	0.114
≥ 3 cm	20(31.75%)	2	18	
EGFR mutations				
Negative	32(50.79%)	14	18	< 0.001
Positive	31(49.21%)	1	30	
K-ras mutations				
Negative	58(92.06%)	13	45	0.585
Positive	5(7.94%)	2	3	

### LAPTM4B is a valuable serum marker in patients with LAC

**Fig. 2:**
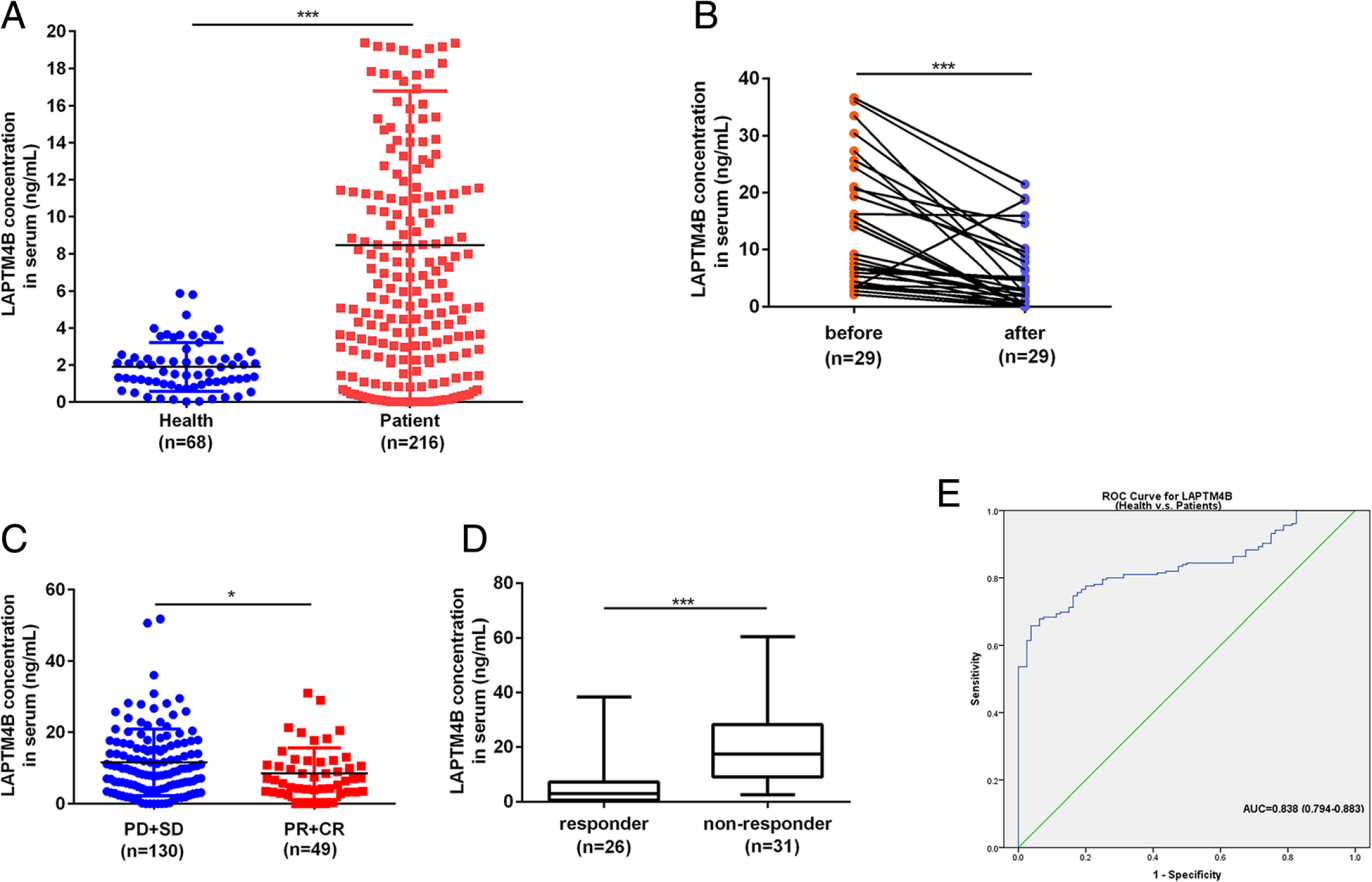
LAPTM4B is a serum marker in patients with lung adenocarcinoma. **a** The concentration of LAPTM4B in the serum of patients with LAC and healthy controls. **b** The changes of LAPTM4B concentration in chemotherapy patients with LAC. **c** Concentration of LAPTM4B in LAC patients of different chemotherapy efficacy groups. **d** LAPTM4B concentration in LAC patients with different EGFR-TKIs sensitivity. **e** Receiver operating characteristic (ROC) curve for LAPTM4B in healthy controls and patients with LAC. *P < 0.05, ***P < 0.001

In order to test the serum LAPTM4B as the potential serological marker for LAC, we first examined the LAPTM4B expression in the serum of a large set of patients with LAC and found that the serum LAPTM4B levels were dramatically increased compared with healthy controls (Table 2, Fig. 2a). Serum LAPTM4B levels of LAC patients were significantly correlated with smoking, advanced clinical stages, lymph node metastasis, anaplastic lymphoma kinase (ALK) rearrangements and EGFR mutations. In contrast, serum LAPTM4B levels displayed no association with gender, age, hypertension, depth of tumor infiltration and distant metastasis (Table 3).

**Table 2:** The concentration of LAPTM4B in the serum of patients with LAC and healthy controls (median(IQR))

Group	Number	LAPTM4B(ng/mL)	*P* value
Patients	216	6.067(9.909)	< 0.001
Controls	68	1.616(1.406)

**Table 3:** Correlation of serum LAPTM4B with clinical indicators in patients with LAC (median(IQR))

Characteristics	Number	LAPTM4B(ng/mL)	*P* value
Gender			
Male	95(43.98%)	9.478(10.410)	0.123
Female	121(56.02%)	7.664(10.349)	
Age			
≤ 60	100(46.30%)	7.695(9.131)	0.201
> 60	116(53.70%)	9.122(11.981)	
Smoking			
No	141(65.28%)	7.394(10.201)	0.019
Yes	75(34.72%)	10.469(11.220)	
Hypertension			
No	157(72.69%)	8.541(11.009)	0.819
Yes	59(27.31%)	8.249(8.749)	
Clinical stages			
I + II	120(55.56%)	7.315(10.454)	0.023
III + IV	96(44.44%)	9.895(10.420)	
Depth of infiltration			
T1 + T2	170(78.70%)	7.944(9.834)	0.079
T3 + T4	46(21.30%)	10.374(10.006)	
Lymph node metastasis			
No	119(55.09%)	7.293(10.323)	0.011
Yes	97(44.91%)	10.225(10.496)	
Distant metastasis			
No	158(73.15%)	8.160(10.278)	0.381
Yes	58(26.85%)	9.283(9.624)	
ALK-ventana			
Negative	179(82.87%)	7.277(8.885)	< 0.001
Positive	37(17.13%)	14.190(6.668)	
EGFR mutations			
Negative	139(64.35%)	6.942(7.532)	0.001
Positive	77 (35.65%)	11.205 (14.330)	

In addition, we evaluated the treatment effects on the serum LAPTM4B levels of LAC patients. The serum LAPTM4B level was significantly decreased after chemotherapy in 29 LAC patients (Table 4, Fig. 2b). We further analyzed the serum LAPTM4B levels of different chemotherapy efficacy groups and the result demonstrated that the serum concentration of LAPTM4B in PD + SD group was higher than PR + CR group (Table 5, Fig. 2c).

**Table 4:** The changes of serum LAPTM4B in patients with LAC before and after chemotherapy (median(IQR))

Group	Number	LAPTM4B (ng/mL)		*P* value
		Before chemotherapy	After chemotheropy	
Chemotherapy patients	29	9.192 (17.807)	2.945 (8.977)	< 0.001

**Table 5:** Comparison of serum LAPTM4B levels in patients with LAC between different chemotherapy efficacy groups (median(IQR))

Efficacy	Number	LAPTM4B(ng/mL)	*P* value
PR + CR	49	6.917 (8.458)	0.039
PD + SD	130	9.819 (12.110)	

In order to investigate the clinical significance of LAPTM4B in EGFR-TKIs sensitivity, we analyzed a cohort of 57 LAC patients with EGFR mutations. These patients had received EGFR-TKIs treatment, either as front line or salvage treatment. As shown in Table 6, Fig. 2d, 26 patients were sensitive to EGFR-TKIs and 31 patients were resistant to EGFR-TKIs. Compared with sensitive patients, a higher proportion of patients who were resistant to EGFR-TKIs treatment exhibited high LAPTM4B levels.

**Table 6:** High serum concentration of LAPTM4B is inversely associated with EGFR-TKI sensitivity in LAC (median(IQR))

EGFR-TKI	Number	LAPTM4B(ng/mL)	*P* value
responder	26	2.860 (6.416)	< 0.001
non-responder	31	17.373 (19.120)	

The approximate area under the Receiver Operating Characteristic (ROC) curve assessing serum LAPTM4B as a diagnostic tool for detection of LAC against normal controls was 0.838 (95% CI:0.794~0.883, *P* < 0.001), at a cut off value of 2.761 ng/mL (Fig. 2e). The sensitivity and specificity were 75.6 and 82.5%, respectively.

Therefore, our results indicated that LAPTM4B may be identified as a valuable serum biomarker for diagnosis and treatment of lung adenocarcinoma.

### LAPTM4B promotes proliferation, migration and invasion of lung adenocarcinoma

**Fig. 3:**
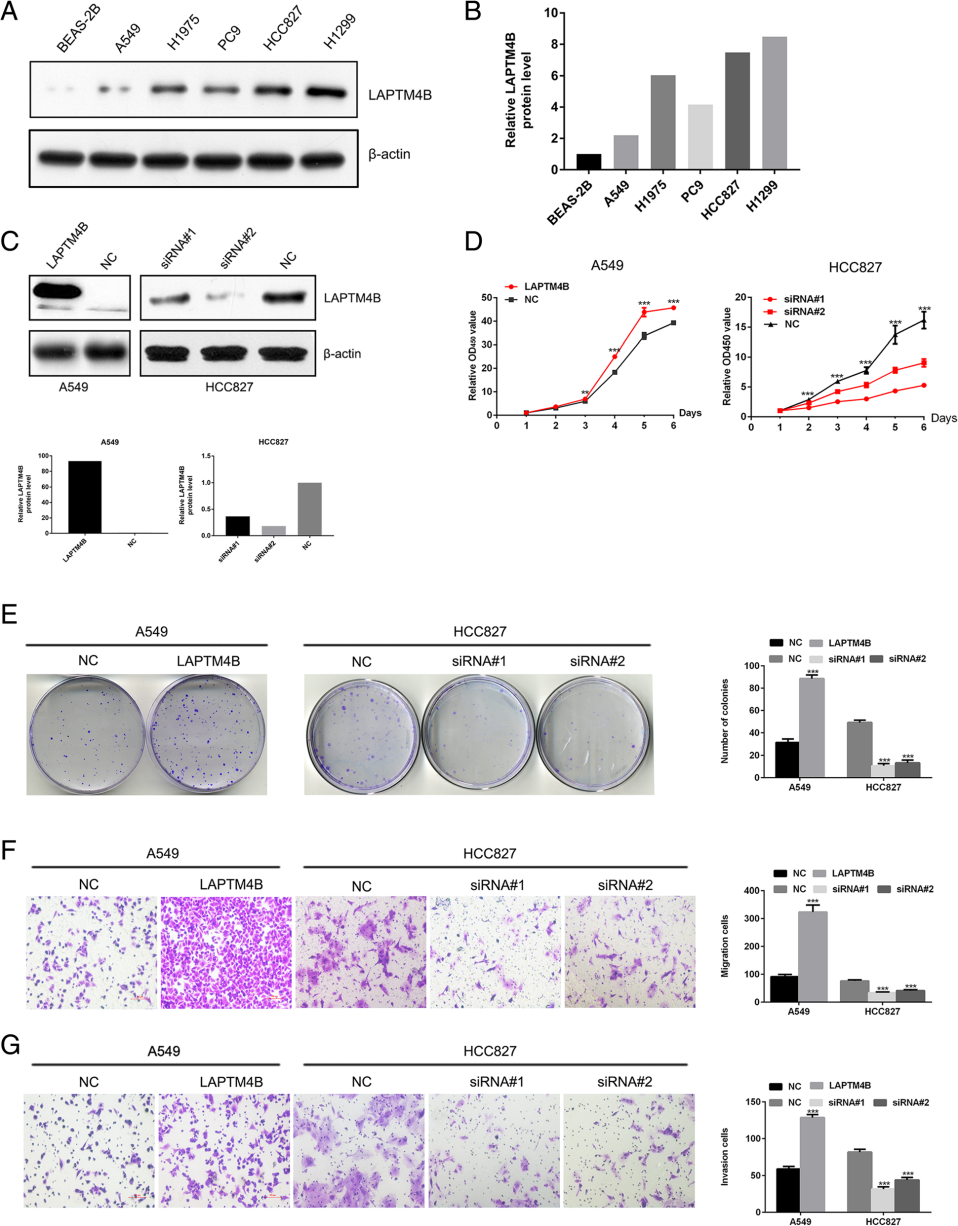
LAPTM4B promotes the proliferation, migration and invasion of LAC cells. **a** Western blotting analysis of LAPTM4B expression in human bronchial epithelial BEAS-2B cells and five LAC cell lines. β-actin was used as a loading control. **b** The protein levels were measured by Image J software. The expression level of LAPTM4B in BEAS-2B was set to 1.0. **c** Cells were infected with LAPTM4B overexpression lentivirus in A549 cells and transfected with specific LAPTM4B siRNAs in HCC827 cells. Endogenous LAPTM4B expression was indicated by the bottom band (35kDA) and the top band (38kDA) represented the exogenous LAPTM4B overexpression. Relative LAPTM4B protein levels were measured by Image J. **d** In CCK-8 assays, overexpression of LAPTM4B significantly increased the growth rate of A549, while downregulation of endogenous LAPTM4B significantly reduced the growth rate of HCC827 cells. Each bar represents the mean values±SD of three independent experiments. **e** Overexpression of LAPTM4B increased, while downregulation of endogenous LAPTM4B reduced, the colony numbers in colony formation assay. Each bar represents the mean values±SD of three independent experiments. **f** and **g** Overexpression of LAPTM4B increased, while downregulation of LAPTM4B reduced, the migration ability (**f**) and invasion ability (**g**) of A549 and HCC827 cells. Each bar represents the mean values±SD of three independent experiments. **P < 0.01, ***P < 0.001

To determine the biological roles of LAPTM4B in LAC, we first observed LAPTM4B expression levels in human bronchial epithelial BEAS-2B cells and five LAC cell lines (A549, H1975, PC9, HCC827 and H1299). BEAS-2B exhibited the lowest expression level of LAPTM4B. A549 showed relatively lower LAPTM4B expression than the other cell lines (Fig. 3a, b). Then, we constructed LAPTM4B stably overexpressing A549 cells by lentivirus infection and endogenously knocking down LAPTM4B in HCC827 cells by specific siRNAs transfection (Fig. 3c). CCK-8 assay revealed that ectopic expression of LAPTM4B significantly increased, while silencing LAPTM4B reduced, the cell proliferation of LAC cells (Fig. 3d). Colony formation assay indicated that upregulation of LAPTM4B enhanced the colony formation abilities of LAC cells. Conversely, downregulation of LAPTM4B decreased the colony formation ability (Fig. 3e).

To investigate the effects of LAPTM4B on the metastatic ability of LAC cells, we performed transwell assays and found that overexpression of LAPTM4B increased migration and invasion ability of A549 cells compared to the controls (Fig. 3f, g). In contrast, knocking down LAPTM4B significantly reduced migration and invasion ability of HCC827 cells (Fig. 3f, g). Collectively, these results indicated that LAPTM4B promotes cell proliferation, migration and invasion of LAC cells.

### PI3K/AKT and epithelial-mesenchymal transition (EMT) signals are involved in the tumor-promoting role of LAPTM4B in LAC

**Fig. 4:**
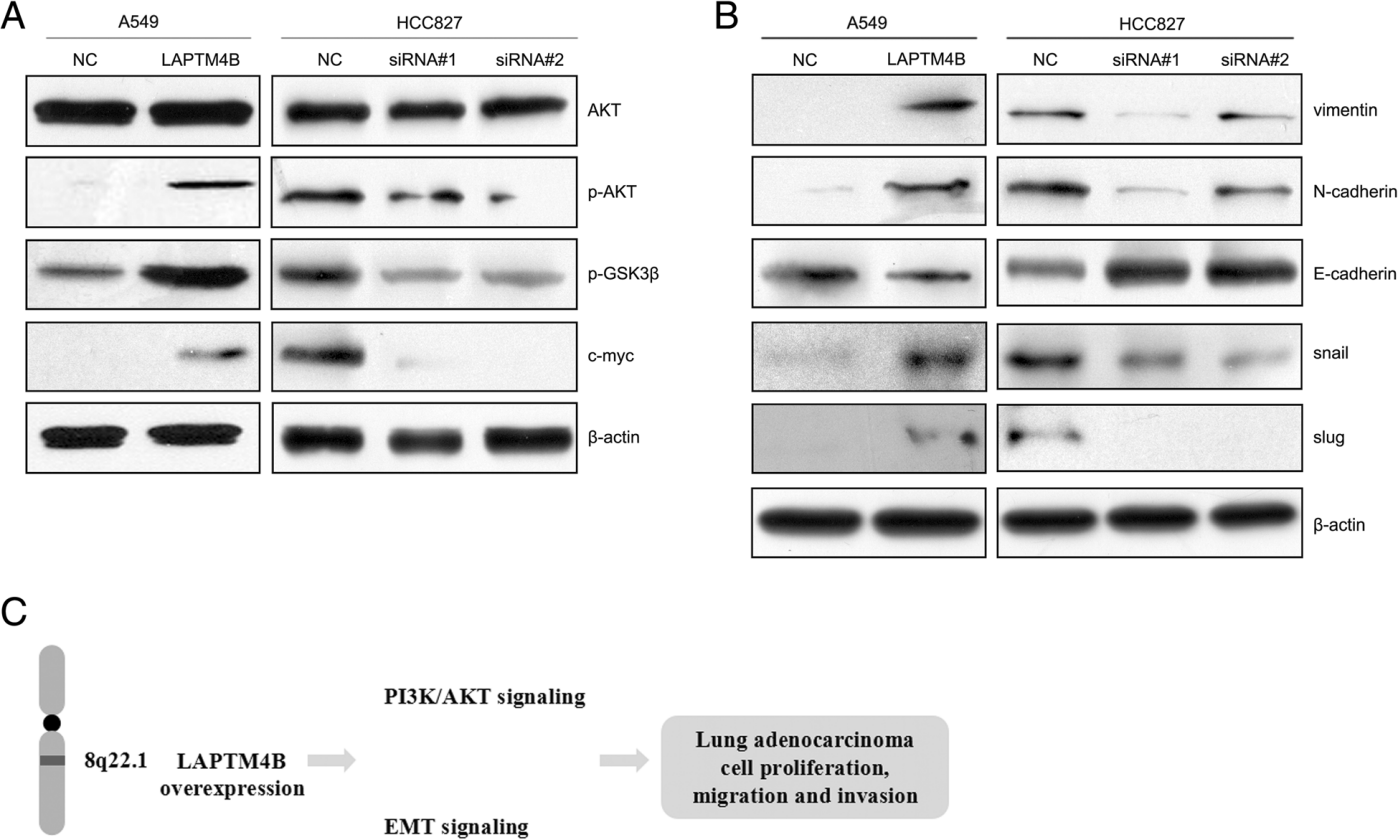
LAPTM4B promotes proliferation, migration and invasion by PI3K/AKT and EMT signals in LAC. **a** Western blotting analysis of PI3K/AKT-associated proteins in the indicated LAC cells. **b** Western blotting analysis of EMT-associated proteins in the indicated LAC cells. **c** Hypothetical model of LAPTM4B in regulation of proliferation, migration and invasion of lung adenocarcinoma cells

To explore the molecular mechanisms underlying LAPTM4B functions as a tumor promoter in LAC, it was overexpressed or knocked down in A549 and HCC827 cells. Western blotting revealed that upregulating of LAPTM4B increased, while silencing LAPTM4B decreased the phosphorylation levels of AKT and GSK3β. However, the total AKT and GSK3β levels were unchanged (Fig. 4a). Moreover, the cycle-related gene c-myc was also increased in LAPTM4B-overexpressing cells but decreased in LAPTM4B-silenced cells. Furthermore, mesenchymal-related genes including vimentin, N-cadherin, snail and slug were also increased concomitant with decreased expression of epithelial-marker E-cadherin in LAPTM4B-overexpressing A549 cells (Fig. 4b). The changes of EMT-related genes were opposite in LAPTM4B-silenced HCC827 cells. Taken together, these results suggested that PI3K/AKT and EMT pathways may mediate the pro-tumor effects of LAPTM4B in LAC cells (Fig. 4c).

### Mutant EGFR influences LAPTM4B levels through PI3K-AKT-mTOR pathway

**Fig. 5:**
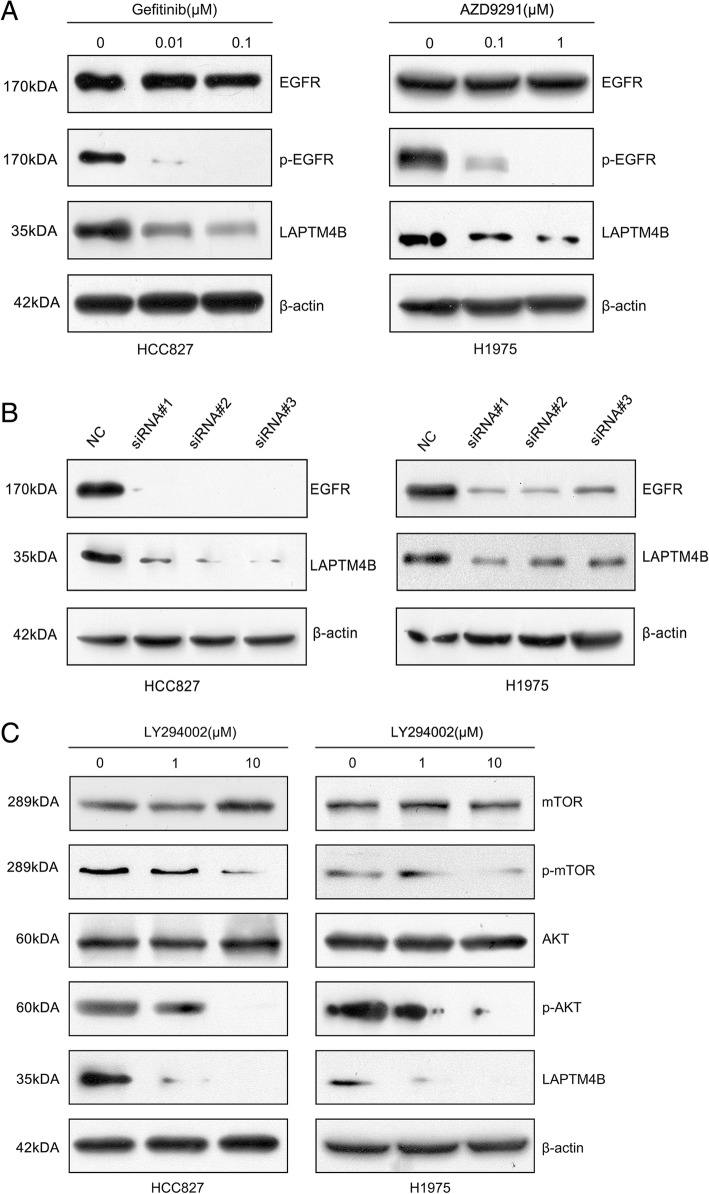
Mutant promotes LAPTM4B expression related to PI3K/AKT signal in LAC cells. **a** Western blotting analysis of LAPTM4B protein expression after dose-dependent treatment with gefitinib in HCC827 and AZD9291 in H1975 cells. **b** Western blotting analyzed the expression levels of LAPTM4B in siRNAs-transfected HCC827 and H1975 cells. **c** Western blotting analysis of LAPTM4B protein levels after dose-dependent treatment with LY294002 in HCC827 and H1975 cells

Interestingly, we noted that EGFR mutation status was associated with LAPTM4B expression levels both in LAC tissues and serum samples (Table 1, Table 3). LAC patients with EGFR mutations exhibited higher LAPTM4B levels compared to those with EGFR wild-type. It is known that the EGFR mutations lead to ligand-independent activation of the EGFR and down stream pathways are highly activated [21]. Thus, we hypothesized if the kinase activity of mutant EGFR could influence LAPTM4B expression in LAC cells. We explored this hypothesis by inhibiting EGFR kinase activity with EGFR-TKIs in HCC827 (exon 19 deletion) and H1975 (L858R point mutaion and T790 M) cells harboring EGFR mutations. The result showed that gefitinib and AZD9291, EGFR tyrosine kinase inhibitors, suppressed LAPTM4B expression in both HCC827 and H1975 cells in a dose-dependent manner, suggesting a positive correlation between LAPTM4B expression and EGFR kinase activation in LAC cells (Fig. 5a). In addition, knockdown of EGFR expression levels by specific siRNAs transfection dramatically reduced LAPTM4B protein levels in HCC827 and H1975 cells (Fig. 5b). The result directly indicated the interaction of LAPTM4B between mutant EGFR.

Next, we explored the signaling pathway components involved in regulating LAPTM4B downstream of mutant EGFR. To determine whether the inactivation of PI3K/AKT signaling was involved in down-regulation of LAPTM4B, HCC827 and H1975 cells were treated with LY294002, a PI3K inhibitor. In Fig. 5c, as expect, LY294002 decreased cellular phospho-AKT and phospho-mTOR protein levels consistent with LAPTM4B in a dose-depend manner in both two cell lines with EGFR mutations. The total AKT and mTOR levels were unchanged. Therefore, these data suggested that mutant EGFR may promote LAPTM4B expression via PI3K/AKT axis.

### LAPTM4B enhances the effects of EGFR on cell proliferation, migration and invasion and inhibits gefitinib-induced cell apoptosis

**Fig. 6:**
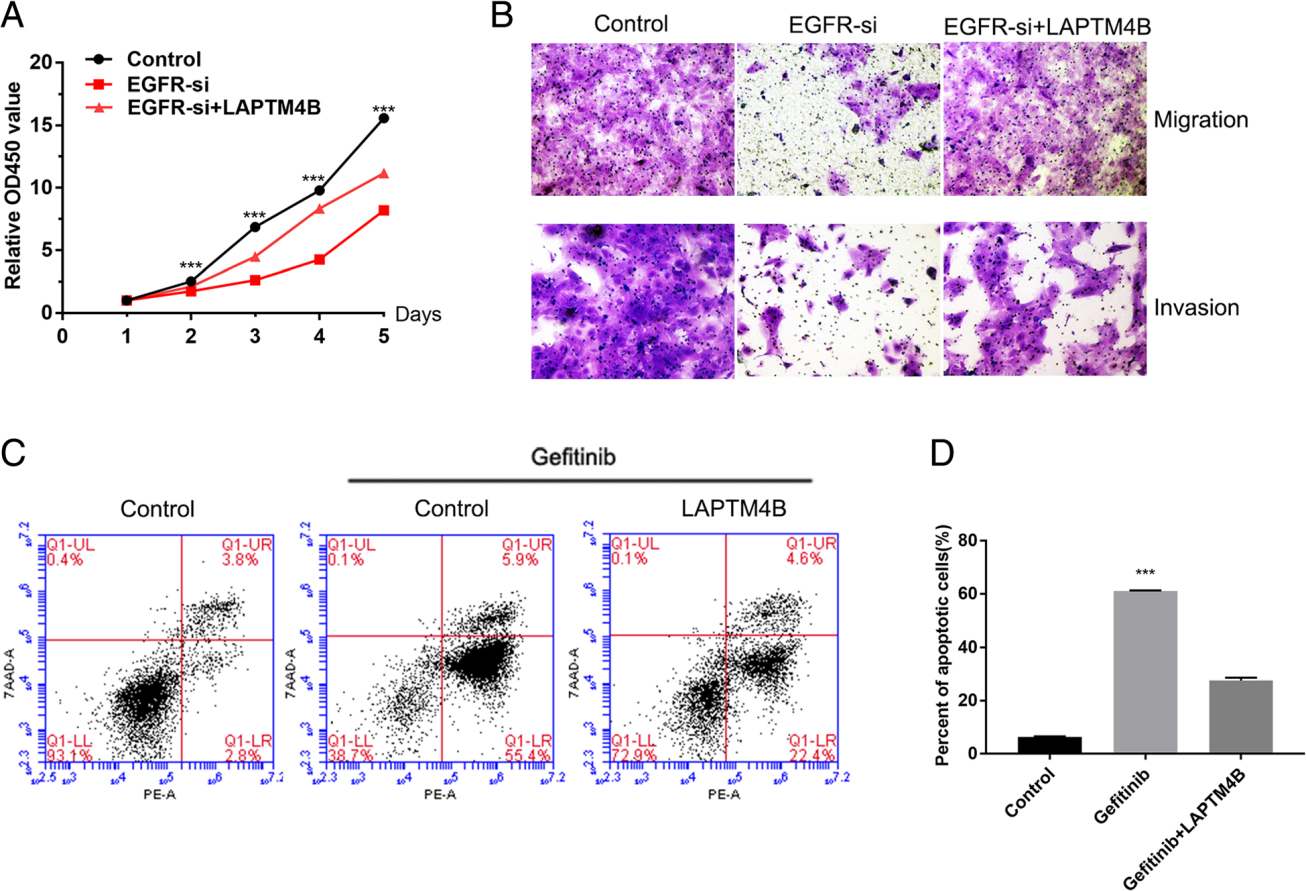
LAPTM4B enhances the effects of EGFR on cell proliferation, migration and invasion and inhibits gefitinib-induced cell apoptosis. **a** HCC827 cells were transfected with EGFR-specific siRNA with or without LAPTM4B overexpression. Cell viability was determined by CCK-8 assays. Control: transfected scrambled vectors, EGFR-siRNA: transfected with EGFR-specific siRNA, EGFR-siRNA+LAPTM4B:transfected with EGFR-specific siRNA and LAPTM4B overexpression vector. **b** The effects of EGFR and LAPTM4B on cell migration and invasion were evaluated by transwell assays. **c** The HCC827 cells were incubated with 0.01 μM gefitinib (Gefitinib group) and DMSO (control group), respectively. After 48 h incubation, cells were harvested and analyzed by FACSCalibur flow cytometer. Each bar represents the mean values±SD of three independent experiments. ***P < 0.001

To further investigate the roles of LAPTM4B in EGFR-mediated LAC progression. We stably overexpressed LAPTM4B but knocked down EGFR using siRNA in HCC827 cells. The CCK-8 assay showed that EGFR siRNA noticeably reduced LAC cell proliferation; however, LAPTM4B overexpression markedly rescued cell proliferation arrest by EGFR knockdown (Fig. 6a). In addition, cells transfected with EGFR siRNA displayed significantly lower ability of migration and invasion compared with controls. Meanwhile, LAPTM4B overexpression dramatically rescued the inhibition of cell migration and invasion induced by EGFR siRNA (Fig. 6b).

Gefitinib selectively binds to the kinase domain and inhibits phosphorylation of EGFR, leading to apoptotic cell death [22–24]. As shown in Fig. 6c and d, gefitinib significantly induced apoptosis of HCC827 cells compared to the controls. However, the effect was also rescued by LAPTM4B restoration, suggesting that LAPTM4B could inhibit the gefitinib-induced apoptosis of HCC827 cells with EGFR mutations.

## Discussion

NSCLC is one of the most common causes of cancer related death worldwide [25]. Patients diagnosed with NSCLC are usually at late stage, and the prognosis is very poor. It’s significant to find an effective indicator to diagnose NSCLC in clinical practice. LAPTM4B was first identified in hepatocellular carcinoma and overexpressed in multiple solid tumors. In addition, LAPTM4B played critical roles in tumorigenesis and tumor metastasis in hepatocellular carcinoma, ovarian cancer, breast cancer and cervical cancer [26–29]. Our recent efforts demonstrated that transcription factor AP4 positively regulates LAPTM4B to promote cell growth, metastasis and chemotherapy resistance in hepatocellular cancer and breast cancer [19, 30]. Based on the previous studies, we explored the biological role and clinical significance of LAPTM4B in lung adenocarcinoma.

In the present study, we sought to characterized LAPTM4B expression in LAC specimens and its impact on oncogenic phenotype of LAC cells and signaling pathways. LAPTM4B expression level was analyzed from TCGA database and found that it was overexpressed in LAC compared to normal tissues. Then, we investigate LAPTM4B protein levels in 63 LAC tissues by IHC and our results revealed that high expression of LAPTM4B correlated with aggressive clinicopathological features (including advanced clinical stages, lymph node metastasis and EGFR mutations). Moreover, our data also identified that OS and PFS of LAC patients were significantly worse in the LAPTM4B high expression group compared to the low expression group and the result was consistent with the previous study reporting that LAPTM4B was associated with poor survival in LAC but not in all NSCLC patients [17].

LAPTM4B is a type III transmembrane protein with four putative transmembrane regions and highly expressed in LAC tissues, so we test whether serum levels of LAPTM4B could be used as a tumor marker in LAC. This is the first report investigating the value of serum LAPTM4B levels as a biomarker for the patients with LAC. Our results demonstrated that serum levels of LAPTM4B were much higher in a relatively large series of LAC samples (*n* = 216) than those in the healthy controls. Furthermore, serum LAPTM4B levels were positively correlated with smoking, advanced clinical stages, lymph node metastasis, ALK rearrangements and EGFR mutations. In addition, we evaluated the value of serum LAPTM4B levels in chemotherapy response of LAC patients. The results showed that serum LAPTM4B levels were significantly decreased after chemotherapy. We also compared LAPTM4B levels in different chemotherapy efficacy groups and found that serum LAPTM4B levels of chemotherapy patients with PD and SD were much higher than those with PR and CR, suggesting LAPTM4B have the potential to predict chemotherapy response of LAC patients. EGFR gene mutations are detected in 30 to 40% of patients with NSCLC in China and associated with poor prognosis [31]. Patients with EGFR mutations are highly sensitive to EGFR-TKIs [32] but approximately 10% of NSCLC patients harboring EGFR mutations exhibit primary resistance [33]. Our study demonstrated that high LAPTM4B expression was associated with EGFR-TKI resistance in LAC patients with EGFR mutations. Additional large-cohort studies are needed to confirm the finding. ROC curve analysis revealed that the sensitivity and specificity of LAPTM4B were 75.6 and 82.5%, respectively. Serum LAPTM4B level showed an early diagnostic performance comparable to CEA and CYFR 21–1 [34].

Our in vitro findings revealed that overexpression of LAPTM4B promoted, while silencing LAPTM4B inhibited proliferation, migration and invasion of LAC cells. To further clarify the mechanism of LAPTM4B involvement in cell growth and migration, the associated proteins of PI3K/AKT and EMT signals in LAC cells were investigated. Western blotting results showed that the two signals were activated in LAPTM4B overexpression cells. It is worthwhile to mention that previous report has shown that the PPRP motif contained in the N-terminus of LAPTM4B-35 could interact with the p85α regulatory subunit of PI3K which results in activation of PI3K/AKT signals. PI3K/AKT signaling pathway participated in LAPTM4B-promoting tumor progression and multidrug resistance in hepatocellular carcinoma, breast cancer and prostate cancer [19, 26, 30, 35]. The current findings along with the previous published reports suggest that LAPTM4B/AKT signaling axis may present a novel target for the treatment of LAC. Additionally, c-myc regulates cell growth, differentiation, apoptosis and motility, and is involved in the pathogenesis of many cancers [36, 37]. In the study, we also found that c-myc protein was significantly increased by LAPTM4B overexpression and may play an important role in LAC progression.

Notably, we found that high LAPTM4B expression was associated with EGFR mutations in LAC tissues and serum samples. Moreover, A549 cell with EGFR-wild type exhibited relatively lower LAPTM4B expression compared to H1299 cell line displaying high invasiveness and other cell lines (H1975, PC9 and HCC827) with EGFR mutations. These observations led us to investigate the association between LAPTM4B expression and EGFR-activating mutations. Of note, activating mutations within the EGFR tyrosine kinase domain including in-frame deletions in exon 19 and L858R point mutations in exon 21 were found to be predictors of clinical response to EGFR-TKIs [38]. Our results showed that EGFR-TKIs significantly decreased LAPTM4B expression in HCC827 (EGFR exon 19 deletion mutation) and H1975 (L858R point mutation and secondary T790 M mutation) cell lines, insinuating that LAPTM4B may be affected by EGFR kinase activity. Similarly, earlier work has demonstrated that EGFR was co-immunoprecipitated with endogenous LAPTM4B and LAPTM4B overexpression correlates with EGFR activation in gastric cancer cells [39]. Moreover, Tan X et al. reported that upon serum starvation, LAPTM4B senses EGFR inactivation at endosomes and selectively forms a complex with inactive EGFR to initiate autophagy [40]. On the other hand, LAPTM4B interacts with Nedd4 through its PY motifs at the LAPTM4B C-tail and further enhance the ubiquitination of Hrs, which could block EGF-stimulated EGFR intraluminal sorting and degradation. Finally, EGFR signaling could be prolonged and exert influence on tumor progression [41]. Here, in vitro assays revealed that LAPTM4B could enhance the effects of EGFR on cell proliferation, migration and invasion. Together with our findings, LAPTM4B may interact with EGFR and play an important role in the pro-survival functions of EGFR in cancer cells.

As we known, activation of the PI3K-AKT-mTOR axis is more robustly induced by mutant EGFR than by wild-type EGFR. Phosphorylated EGFR being the docking site of PI3K stimulates the activation of AKT [42], and then activates the downstream target molecule mTOR to induce the expression of cell cycle-related proteins and promote cells G1-S transition [43]. Then we further delineated the role of this signaling pathway in regulating LAPTM4B by mutant EGFR. Our result revealed that LY294002, the PI3K inhibitor, could suppress LAPTM4B expression in LAC cells with EGFR mutations. Moreover, previous studies confirmed that EGFR-TKIs can block the PI3K/AKT signaling pathway by inhibiting the kinase activity of mutant EGFR, which have been implicated in the inhibition of cell apoptosis and the promotion of cell growth and motility [44, 45]. However, among patients with advanced EGFR*-*mutated NSCLC, treatment with EGFR-TKIs (e.g., gefitinib, erlotinib, and afatinib) is associated with response rates of 56 to 74% and a median progression-free survival of 10 to 14 months. In addition, the majority of patients will have disease progression within 1 to 2 years after treatment initiation due to the acquired resistance [46–49]. Our results showed that LAPTM4B overexpression could significantly inhibit cell apoptosis induced by gefitinib and make HCC827 cell line harboring EGFR mutations more resistant to EGFR-TKIs. Thus, it is plausible to propose that targeting LAPTM4B and PI3K/AKT pathway may help augment the effects of EGFR-TKIs treatment.

There are several potential limitations in our study. The relative small size population were enrolled in the study, and larger collaborative studies needed to validate our report. In addition, the roles of LAPTM4B on the efficacy of EGFR-TKIs treatment in LAC should be further elucidated.

## Conclusion

In summary, our results revealed that LAPTM4B was overexpressed in LAC and high LAPTM4B expression indicated poor prognosis in patients with LAC. The expression level of LAPTM4B in the serum of patients with LAC is significantly associated with the tumor progression and treatment effects, suggesting that detection of serum LAPTM4B will facilitate the diagnosis and better predict treatment response. Further functional assay indicated LAPTM4B plays an important role in tumor proliferation and metastasis in LAC. In addition, LAPTM4B may take part in EGFR-mediated cell pro-survival and gefitinib resistance of LAC cells with EGFR mutations. These fruitful works indicate that LAPTM4B might be an important biomarker and a novel therapeutic target for LAC.

## Additional files

Additional file 1: **Table S1.** Antibodies used in Western blot assay. (DOCX 13 kb)

Additional file 2: **Table S2.** The sequence for RT-PCR primers and siRNAs. (DOCX 15 kb)

## Abbreviations

ALK: Anaplastic lymphoma kinase
CEA: Carcinoembryonic antigen
CYFRA21-1: Cytokeratin 19 fragment
EGFR: Epidermal growth factor receptor
EGFR-TKIs: Epidermal growth factor receptor-tyrosine kinase inhibitors
ELISA: Enzyme linked immunosorbent assay
EMT: Epithelial-to-mesenchymal transition
HCC: Hepatocellular carcinoma
IHC: Immunohistochemistry
LAC: Lung adenocarcinoma
LAPTM4B: Lysosomal-associated protein transmembrane-4 beta
NSCLC: Non-small cell lung cancer
NSE: Neuron-specific enolase
ProGRP: Pro-gastrin-releasing peptide
ROC: Receiver operating characteristic
