# Supplementary material for: LAPTM4B is a novel diagnostic and prognostic marker for lung adenocarcinoma and associated with mutant EGFR
Source: BMC Cancer. 2019 Apr 2;19:293. doi: 10.1186/s12885-019-5506-7 (PMC6444825; doi:10.1186/s12885-019-5506-7)
Supplement: Supplementary file 1 — Table S1. Antibodies used in Western blot assay. (DOCX 13 kb) [file 12885_2019_5506_MOESM1_ESM.docx]

**Table S1.** Antibodies used in Western blot assay.

| **Antibody** | **Company** | **Catalog** | **Dilution** | **Source** |
| --- | --- | --- | --- | --- |
| p-EGFR | Cell Signaling | 3777 | 1:1000 | Rabbit |
| EGFR | Cell Signaling | 2085 | 1:1000 | Rabbit |
| p-mTOR | Cell Signaling | 5536 | 1:1000 | Rabbit |
| mTOR | Cell Signaling | 2983 | 1:1000 | Rabbit |
| p-AKT | Cell Signaling | 4060 | 1:1000 | Rabbit |
| AKT | Cell Signaling | 9272 | 1:1000 | Rabbit |
| LAPTM4B | Abgent | AP20870a | 1:1000 | Rabbit |
| β-actin | Sigma | A5441 | 1:10000 | Mouse |
