# Supplementary material for: LAPTM4B is a novel diagnostic and prognostic marker for lung adenocarcinoma and associated with mutant EGFR
Source: BMC Cancer. 2019 Apr 2;19:293. doi: 10.1186/s12885-019-5506-7 (PMC6444825; doi:10.1186/s12885-019-5506-7)
Supplement: Supplementary file 2 — Table S2. The sequence for RT-PCR primers and siRNAs. (DOCX 15 kb) [file 12885_2019_5506_MOESM2_ESM.docx]

**Table S2.** The sequence for RT-PCR primers and siRNAs.

| **Name** |  | **Sequence (5’-3’)** |
| --- | --- | --- |
| GAPDH | Sense | ACGGATTTGGTCGTATTGGGCG |
|  | Anti-sense | CTCCTGGAAGATGGTGATGG |
| LAPTM4B | Sense | AAGACCATTAGAAAGCACCAGG |
|  | Anti-sense | ACCAATCTAGGGCAGAACACTTA |
| LAPTM4B-siRNA#1 |  | CTACCTGTTTGGTCCTTAT |
| LAPTM4B-siRNA#2 |  | CAGAGATGATGTCATGTCAGTGAAT |
| EGFR-siRNA#1 |  | GAGGAAATATGTACTACGA |
| EGFR-siRNA#2 |  | GGAGCGAATTCCTTTGGAA |
| EGFR-siRNA#3 |  | GCAGAGGAATTATGATCTT |
